# Effectiveness of the Boston University Approach to Psychiatric Rehabilitation in Improving Social Participation in People With Severe Mental Illnesses: A Randomized Controlled Trial

**DOI:** 10.3389/fpsyt.2020.571640

**Published:** 2020-09-23

**Authors:** Sarita A. Sanches, Wilma E. Swildens, Barbara Schaefer, Mirjam Moerbeek, Talitha L. Feenstra, Antoinette D. I. van Asselt, Unna N. Danner, Jaap van Weeghel, Jooske T. van Busschbach

**Affiliations:** ^1^ Phrenos Center of Expertise for Severe Mental Illness, Utrecht, Netherlands; ^2^ Tilburg School of Social and Behavioral Sciences, Tranzo Scientific Center for Care and Welfare, Tilburg University, Tilburg, Netherlands; ^3^ Altrecht Institute for Mental Health Care, Department Research and Monitoring, Utrecht, Netherlands; ^4^ Inholland University of Applied Sciences, Interprofessional Mental Health Care, Department of Nursing, Amsterdam, Netherlands; ^5^ Parnassia Psychiatric Institute, The Hague, Netherlands; ^6^ Department of Methodology and Statistics, Utrecht University, Utrecht, Netherlands; ^7^ University of Groningen, University Medical Center Groningen, Department of Epidemiology, Groningen, Netherlands; ^8^ University of Groningen, Groningen Research Institute of Pharmacy, Groningen, Netherlands; ^9^ Centre for Nutrition, Prevention, and Health Services Research, Institute for Public Health and the Environment (RIVM), Bilthoven, Netherlands; ^10^ University of Groningen, University Medical Center Groningen, Department of Health Sciences, Groningen, Netherlands; ^11^ Altrecht Eating Disorders Rintveld, Altrecht Mental Health Institute, Zeist, Netherlands; ^12^ University of Groningen, University Medical Center Groningen, University Center of Psychiatry, Rob Giel Onderzoekcentrum, Groningen, Netherlands; ^13^ School of Human Movement and Education, Windesheim University of Applied Sciences, Zwolle, Netherlands

**Keywords:** severe mental illnesses, social participation, psychiatric rehabilitation, paid employment, unpaid employment, education, meaningful daily activities

## Abstract

**Background:**

People with severe mental illnesses (SMIs) have difficulty participating in society through work or other daily activities.

**Aims:**

To establish the effectiveness with which the Boston University Approach to Psychiatric Rehabilitation (BPR) improves the level of social participation in people with SMIs, in the Netherlands.

**Method:**

In a randomized controlled trial involving 188 people with SMIs, we compared BPR (n = 98) with an Active Control Condition (ACC, n = 90) (Trial registration ISRCTN88987322). Multilevel modeling was used to study intervention effects over two six-month periods. The primary outcome measure was level of social participation, expressed as having participated in paid or unpaid employment over the past six months, as the total hours spent in paid or unpaid employment, and as the current level of social participation. Secondary outcome measures were clients’ views on rehabilitation goal attainment, Quality of Life (QOL), personal recovery, self-efficacy, and psychosocial functioning.

**Results:**

During the study, social participation, QOL, and psychosocial functioning improved in patients in both groups. However, BPR was not more effective than ACC on any of the outcomes. Better social participation was predicted by previous work experience and a lower intensity of psychiatric symptoms.

**Conclusions:**

While ACC was as effective as BPR in improving the social participation of individuals with SMIs, much higher percentages of participants in our sample found (paid) work or other meaningful activities than in observational studies without specific support for social participation. This suggests that focused rehabilitation efforts are beneficial, irrespective of the specific methodology used.

## Introduction

Severe mental illnesses (SMIs) have an enormous impact on people’s daily lives and social participation (1). Common problems include unemployment rates that range from 65% to 93% (2), and difficulties with other daytime activities such as education, unpaid employment, or activities outside the home (3, 4). This is a serious issue, not only because social participation is an important facilitator of many definitions of recovery but also because it enhances financial independence and promotes Quality of Life (QOL) (4, 5). Earlier studies have shown that 55%–96% of people with SMIs have an explicit wish to improve their social participation (6, 7).

Fortunately, social participation is now a key objective of the mental health care (MHC) services responsible for people with SMIs (4). Psychiatric rehabilitation methods could help people with SMIs increase their social participation, and as a consequence, support them in their recovery process by helping them lead meaningful lives (8). One evidence-based method that helps them obtain and keep paid employment is Individual Placement and Support (IPS) (9). Methods that provide support in multiple life domains include Illness Management and Recovery (IMR) (10), the Strengths model (11), and the Boston University Approach to Psychiatric Rehabilitation (BPR) (12). The wider scope of these approaches can be particularly beneficial for individuals who are unwilling or incapable of paid employment, and thus pursue unpaid work or meaningful daytime activities. The aim of BPR is to “help persons with psychiatric disabilities increase their ability to function successfully and be satisfied in the environment of their choice with the least amount of ongoing professional intervention” (12). BPR uses a well described systematic methodology that distinguishes four phases: 1) Exploring the patient’s goals in the near (6 months to 2 years) future in a self-chosen rehabilitation area (housing, work, education, and social contacts); 2) Choosing a specific goal, making a plan for necessary support and skills to develop to realize this goal; 3) Getting the goal, realizing the plan, learning skills, and organizing support; and 4) Keeping the attained goal (12, 13). When goal setting is a problem, the readiness of patients for rehabilitation is further explored and developed. Since an important component of BPR is helping patients explore their options, they do not need to have a clearly defined idea or plan for change in order to receive BPR. Therefore, the approach may be particularly suited to those who have been living with mental illness for a long time and have lost confidence in their own ability to initiate change. Earlier Randomized Controlled Trials (RCTs) on the effectiveness of BPR showed positive effects regarding social participation, social contacts, and the attainment of self-chosen rehabilitation goals (14–16). A prospective study (15) and an RCT (17) also found that BPR positively influenced QOL, psychiatric symptoms, empowerment, psychosocial functioning, and needs for care. However, in a study focused on paid employment, Rogers et al. (18) found no difference between BPR and a control condition.

As BPR is a well-implemented rehabilitative approach in the Netherlands, and as the study by Swildens et al. (14) showed that it produced promising results regarding social participation, we investigated its effectiveness in a large group of people with SMIs who wished to improve their social participation. We therefore compared BPR with an “active” control condition (ACC) in which mental health practitioners who had not been trained in BPR were given clear instructions to proactively offer support with rehabilitation goals. We hypothesized that BPR would be more effective than ACC with regard not only to increasing the social participation of individuals with SMIs but also to improving their subjective QOL, personal recovery, self-efficacy, psychosocial functioning, and to attaining subjective rehabilitation goals.

## Methods

### Design

The study design, methods, and analysis plan are described in detail in Sanches et al. (19) In brief, from 2014 to 2017, we conducted a multicenter two-parallel-arm RCT with repeated measures at baseline and at 6 and 12 months (University Medical Center Groningen ethical approval reference number 2013/70; trial registration ISRCTN88987322). Randomization was conducted by an independent researcher, and participants were stratified by center and previous work experience. Data were collected by trained interviewers blinded to treatment allocation.

### Participants

Participants all had SMIs and were drawn from various rural and urban regions of the Netherlands. They were recruited directly through posters and information leaflets in MHC waiting rooms and also through case-managers, psychiatrists, and nurses. Inclusion criteria were a) having a diagnosis of SMI (a DSM-IV or DSM-5 diagnosis, long-term contact with services, and functional impairments that substantially interfered with or limited major life activities); b) having expressed a wish for change in social participation; and c) age 18–64. Although hospitalization during enrolment was an exclusion criterion, we did not exclude participants with enduring comorbid eating disorders, who were often hospitalized due to low bodyweight but were otherwise able and eager to participate. To ensure that these participants were distributed evenly between interventions, they were stratified for hospitalization in the 6 months before inclusion (yes/no) and for length of stay (less/more than 3 months). Thirteen individuals with eating disorders were included, 10 of whom had been hospitalized in the 6 months before enrollment. Written informed consent was obtained from all participants.

### Interventions

#### BPR

BPR was designed to help individuals with SMIs achieve and retain goals in four rehabilitation domains: housing, education, work, and social contacts. Such goals include wanting a certain number of hours of paid administrative work, or working 1 day a week as a volunteer in a care home.

BPR comprises four phases: exploring rehabilitation goals, choosing them, getting them, and keeping them (20). While the rehabilitation process is facilitated by a practitioner, the goal and the pace are directed by the person with SMI. No clearly predefined goal is required in order to start.

In this study, BPR was delivered by 28 trained social workers, nurses or employment specialists who had completed additional training in BPR. Participants were offered at least one session every 2 weeks. There was no predetermined total number of sessions. BPR treatment fidelity was assessed retrospectively by independent BPR experts using the Fidelity of Rehabilitation instrument (FiRe) on a scale from 1 (lowest level of model adherence) to 5 (highest level) (21). Fidelity scores were calculated for a random selection of two-thirds of BPR processes.

#### ACC

Participants in the ACC condition were also offered at least one session every 2 weeks by one of 55 practitioners who had backgrounds similar to those of the BPR practitioners, but lacked training in BPR. Due to the heterogeneous nature of this condition, measuring fidelity was not applicable.

BPR and ACC practitioners alike were offered regular peer coaching and were allowed to involve additional inputs such as specialized vocational services.

### Primary Outcome Measures

To measure self-reported social participation over two periods of six months, we used 1) the dichotomized score on the Occupation and Employment subscale of the Birchwood Social Functioning Scale (SFS_OE) (22), i.e., unemployed (0–6) vs. employed (7–10); and 2) the total number of hours spent in paid and unpaid employment. To rate the current level of social participation, the primary clinician used the Dutch National Societal Participation Ladder, which establishes the level of social participation in six steps ranging from severe social isolation to regular paid employment (23).

### Secondary Outcome Measures

All secondary outcomes were self-report measures. As in previous studies (14), goal attainment was conservatively dichotomized as no goal attainment (no/partial goal attainment) vs. goal attainment (complete goal attainment). At 6 months, goals formulated at baseline were checked for possible changes. After adjustment, the new goal was used as the goal to be evaluated. Subjective psychosocial functioning was measured using the total score of the Birchwood Social Functioning Scale (SFS) (22). For QOL, the total score on the 12 subjective items of the Manchester Quality of Life Schedule (MANSA) (24) was used. Personal recovery was measured using the 41-item Recovery Assessment Scale (RAS) (25), and self-efficacy with the 10-item General Self Efficacy Scale (GSES) (26).

### Other Measures

Sociodemographic information was gathered from participants at baseline and updated at 6 and 12 months. The primary clinician used the symptoms and disabilities version of the Global Assessment of Functioning Scale (GAF-SD) (27) to describe overall psychological, social, and occupational functioning and the extended 24-item Brief Psychiatric Rating Scale (BPRS) (28) to measure psychiatric symptoms and remission. The quality of the therapeutic relationship was measured using the patient and therapist versions of the Helping Alliance Scale (29). Process data on the number of contacts and the use of additional inputs were gathered from practitioners and participants.

### Statistical Analysis

Data were analyzed according to the “intention to treat principle.” (30) Depending on the questionnaire guidelines, person-mean imputation was used when ≤10 or 20% of answers were missing. In other cases, no values were imputed. If patient answers on the primary outcomes were missing, information from the primary clinician or clinical files were used as proxies (12.8% of cases with missing values on ≥1 primary outcomes at 6 months and 8% at 12 months). Descriptive statistics were performed in SPSS-25. Intervention effects were analyzed using 3-level mixed models (HLM3) in HLM v7 (31) with a significance level of α = .05 (two-sided). Level 1 (time expressed as time of measurement; baseline, 6-month follow-up and 12-month follow-up) was nested in subjects (level 2), who were nested in practitioners (level 3). Continuous outcomes were analyzed using a linear multilevel model, full maximum likelihood estimation (ML), and an unstructured (UN) covariance structure, which means that there were no constraints for the variances and covariances (32). Ordinal outcomes were treated as continuous if there were observations in each of at least five categories, and if residuals were distributed normally. Dichotomous outcomes were analyzed with a logistic multilevel model with adaptive Gaussian iterations using 100 iterations and 20 quadrature points. The basic model included time (baseline, 6 months, 12 months); condition (BPR, ACC); and the time x condition interaction. Analyses were controlled for variables that differed between the conditions at baseline (years of practitioner work experience in MHC) and for a small number of possible but essential confounders: baseline level of psychiatric symptoms, previous paid employment (yes/no) (33), living in sheltered housing (yes/no), and use of additional inputs during the study. For all outcome variables, the intercept-only model was used to calculate the intraclass correlations explaining proportions of variance for each level (34). The final model was used to calculate total explained variance (R^2^) for dichotomous outcomes and continuous outcomes (34, 35). Finally, per-protocol analyses were conducted. In the ACC and BPR groups, these included subjects with at least 3 contacts. In the BPR group, they also included a fidelity assessment, in which a minimal score of 3.5 indicated sufficient BPR model fidelity (21).

## Results

Ninety-eight participants were assigned to BPR and 90 to ACC (for characteristics see **Table 1**). Although the groups did not differ with regard to patient and practitioner characteristics and to outcome measures at baseline, ACC practitioners were significantly more experienced than BPR practitioners (p = 0.007).

**Table 1:** Baseline characteristics of study participants and MHC workers, and differences between BPR and ACC groups.

Variable	Total group (188)	BPR(98)	ACC(90)	Test statistic (df)	p
Gender, n (%)					
Male	109 (58)	58(59.2)	51 (56.7)	*χ^2^*(1) = 0.122	0.727
Female	79 (42)	40 (40.8)	39 (43.3)		
Age, years: mean (SD)	39.89 (11.34)	39.18 (10.68)	40.67 (12.04)	t(186) = 0.895	0.372
Main diagnosis, n (%)					
Psychotic disorder	113 (60.1)	59 (60.2)	54 (60)	*χ^2^*(5) = 2.946	0.708
Bipolar disorder	6 (3.2)	2 (2.0)	4 (4.4)		
Depressive or anxiety disorder	13 (6.9)	7 (7.1)	6 (6.7)		
Personality disorder	12 (6.4)	8 (8.2)	4 (4.4)		
Eating disorder	13 (6.9)	8 (8.2)	5 (5.6)		
Other	31 (16.5)	22 (22.4)	22 (24.4)		
Psychiatric symptoms^1^: mean (SD)	44.67 (12.86)	44.25 (13.18)	45.12 (12.56)	t(184) = 0.458	0.647
Duration in MHC (in years): mean (SD)	15.56 (10.76)	15.75 (9.93)	15.36 (11.63)	t(182) = −0.244	0.808
GAF Symptom score^2^: mean (SD)	58.27 (13.26)	58.31 (12.16)	58.22 (14.41)	t(184) = −0.046	0.963
GAF Handicap score^3^: mean (SD)	56.63 (14.89)	56.25 (13.19)	57.03 (16.58)	t(169.91) = 0.355	0.723
Educational level, n (%)^4^ Low^5^ Medium^6^ High^7^	75 (39.9) 79 (42) 33 (17.6)	40 (40.8) 40 (40.8) 17 (17.3)	35 (38.9) 39 (43.3) 16 (17.8)	*χ^2^*(3) = 1.038	0.792
Current daytime activities, n (%) Paid employment Unpaid work Education	13 (7) 68 (36.2) 8 (4.3)	8 (8.2) 33 (33.7) 3 (3.1)	5 (5.6) 35 (38.9) 5 (5.6)	*χ^2^*(1) = 0.467 *χ^2^*(1) = 0.553 *χ^2^*(1) = 0.716	0.494 0.457 0.397
Supported and sheltered housing, n (%) No Yes	146 (77.7) 42 (22.3)	77 (78.6) 21 (21.4)	69 (76.6) 21 (23.3)	*χ^2^*(1) = 0.098	0.754
Paid or unpaid employment over the past six months,^8^ n (%) No Yes	97 (51.6) 91 (48.4)	51 (52.0) 47 (48.0)	46 (51.1) 44 (48.9)	*χ^2^*(1) = 0.016	0.899
Participation ladder^9^: mean (SD)	3.06 (1.08)	3.00 (1.09)	3.12 (1.07)	t(184) = 0.773	0.440
Hours in paid employment:^10^ mean (SD)	13.92 (9.94)	12.88 (8.39)	15.60 (12.93)	t(11) = 0.465	0.651
Hours in unpaid work^11^: mean (SD)	10.11 (7.28)	9.77 (6.57)	10.43 (7.97)	t(66) = 0.370	0.713
QOL^12^: mean (SD)	52.25 (12.11)	52.21 (13.03)	52.21 (11.08)	t(186) = 0.056	0.955
Personal recovery^13^:mean (SD)	84.93 (12.54)	84.42 (12.57)	85.49 (12.56)	t(185) = 0.582	0.561
Self-efficacy^14^: mean, (SD)	28.47 (5.92)	28.06 (5.77)	28.93 (6.08)	t(184) = 1.004	0.317
Psychosocial functioning^15^: mean (SD)	126.54 (21.87)	125.26 (20.16)	127.92 (23.62)	t(186) = 0.833	0.406
Practitioners’ work experience (years): mean (SD)	17.78 (11.19)	15.66 (9.75)	20.09 (12.22)	t(170.11) = 2.736	0.007**
Practitioners’ educational level, n (%)					
Medium	9 (4.8)	3 (3.1)	6 (6.7)	*χ^2^*(1) = 1.338	0.247
High	179 (95.2)	95 (96.9)	84 (93.3)		

*P < 0.05, **P < 0.01, ***P < 0.001.

**Figure 1:**
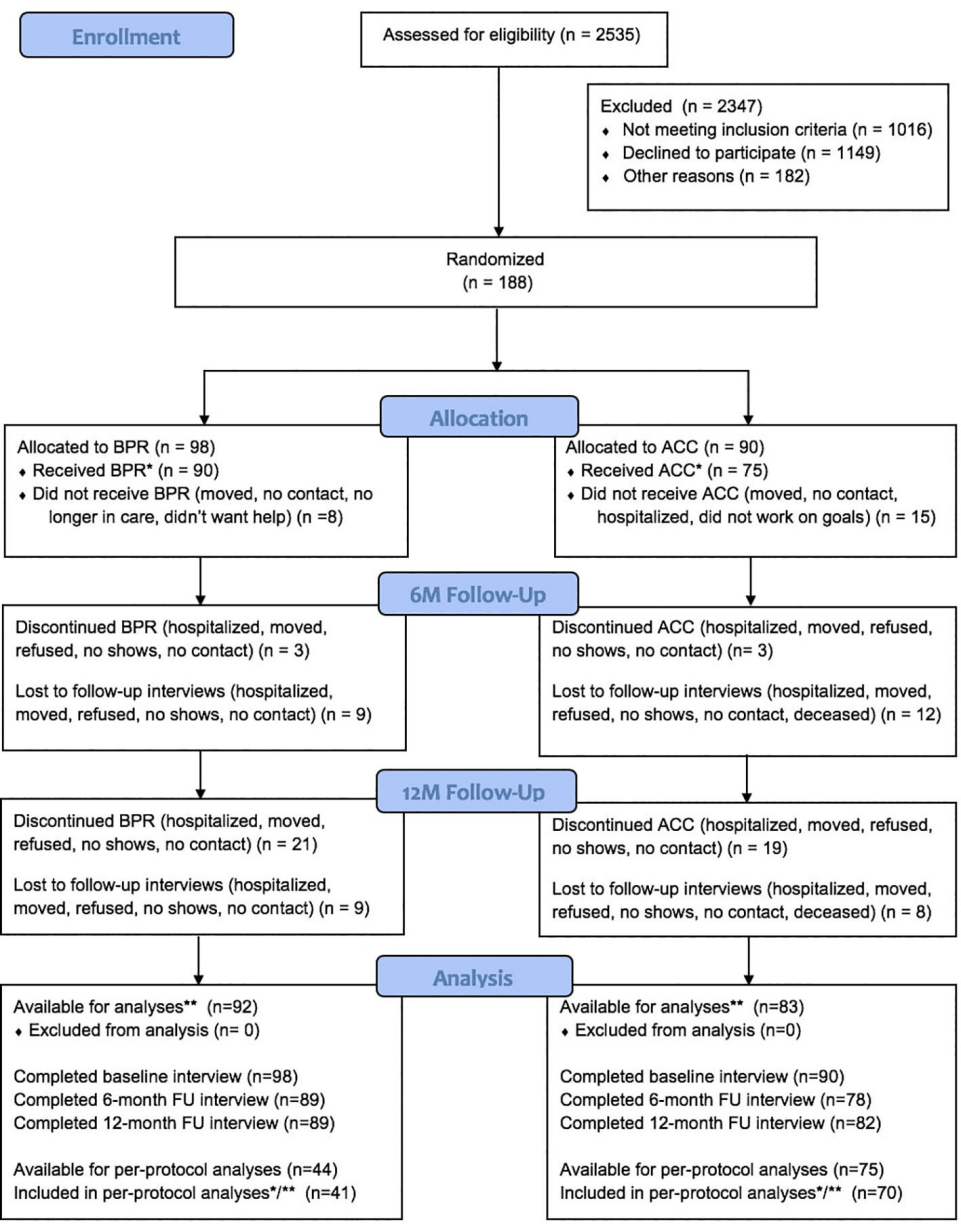
CONSORT flow chart. * Received at least 3 sessions. ** Participants with baseline and 6-month or baseline and 12-month measurement, or all measurements (in multilevel modeling, all available data on outcome are used).

**Figure 1** shows the CONSORT participant flow chart. Drop-out was low and was also comparable between the conditions (12 months: BPR = 9.2%; ACC = 8.9%). While individuals who were lost to follow up did not differ from those with complete data, psychosocial functioning was significantly poorer in dropouts (t = 2.26, df = 186, p = 0.025).

The interventions were well received as 75% of all study participants would recommend the help they received to others (12 months: BPR = 76.3%; ACC = 71.4%, p = 0.138). **Table 2** lists information on goal areas and the rehabilitation process. Almost 50% of participants had paid employment as their initial goal area, followed by unpaid work, education, and other daily activities and social contacts. This was similar between the conditions. In the first 6 months, 20 individuals adjusted their primary goal and goal area, mainly from paid employment to unpaid employment or other meaningful daily activities. Most individuals were nonetheless supported in achieving their goals regarding paid employment. Significantly more participants in ACC than in BPR received additional support (30% versus 15.3% see **Table 2**), which was provided by inputs such as a job coach, through participation in projects organized by local governments, or through IPS. IPS was received by five individuals in ACC but none in BPR.

**Table 2:** Information on goal areas and the rehabilitation process.

Variable	Total group (188)	BPR(98)	ACC(90)	Test statistic (df)	p
Initial goal area, n (%)					
Paid employment	92 (48.9)	44 (44.9)	48 (53.3)	*χ^2^*(4) = 5.404	.248
Unpaid employment	48 (25.5)	24 (24.5)	24 (26.7)		
Education	29 (15.4)	16 (16.3)	13 (14.4)		
Other daily activities and social contacts	16 (8.5)	11 (11.2)	5 (5.6)		
Goal area adjusted in first 6 months, n (%)					
No	167 (89.3)	87 (88.8)	80 (89.9)	*χ^2^*(1) = 0.060	.806
Yes	20 (10.7)	11 (11.2)	9 (10.1)		
Supported goals, n (%)					
Paid employment	84 (45.4)	39 (41.1)	45 (50)	*χ^2^*(3) = 5.371	.147
Unpaid employment	53 (28.6)	26 (27.4)	27 (30)		
Education	31 (16.8)	17 (17.9)	14 (15.6)		
Other daily activities and social contacts	17 (9.2)	13 (13.7)	4 (4.4)		
Number of sessions, mean^16^ (SD)	15.06 (12.11)	16.15 (11.37)	13.87 (12.81)	t(186) = −1.30	0.197
Additional vocational support, n (%)					
No	145 (77.1)	82 (83.7)	63 (70.0)	*χ^2^*(2) = 6.59	0.037*
Yes	42 (22.3)	15 (15.3)	27 (30.0)		
Individual Placement and Support (IPS), n (%)					
No	183 (97.3)	98 (100)	85 (94.4)	*χ^2^*(1) = 5.59	0.018*
Yes	5 (2.7)	0 (0)	5 (5.6)		
Therapeutic relationship at 6-month FU (patient’s perspective)^17^, mean (SD)	40.21 (8.93)	41.79 (7.12)	38.46 (10.35)	t(129.40) = −2.33	0.021*
Therapeutic relationship at 6-month FU (practitioner’s perspective)^18^, mean (SD)	36.64 (4.56)	35.91 (4.92)	37.54 (3.92)	t(158) = 2.28	0.024*
Therapeutic relationship at 12-month FU (patient’s perspective)^17^, mean (SD)	39.54 (9.96)	40.95 (8.54)	38.00 (11.16)	t(130.83) = -1.79	0.075
Therapeutic relationship 12-month FU (practitioner’s perspective)^18^, mean (SD)	36.86 (4.93)	36.64 (4.87)	37.10 (5.01)	t(140) = 0.56	0.577

*P < 0.05, **P < 0.01, ***P < 0.001.

At 6 months, participants in the BPR condition rated the quality of the therapeutic relationship more highly than participants in the ACC condition did. The practitioners’ ratings were the opposite, with ACC practitioners giving higher ratings than BPR practitioners. At 12 months, none of these differences were significant. BPR fidelity was insufficient for one-third (33.8%) of the BPR practitioners assessed (score < 3.5).

### Multilevel Analyses of Primary Outcome Measures

**Table 3** presents the mixed models for all primary outcome measures separately. The SFS_OE subscale significantly improved during the study period **(**t-ratio = 2.30, df = 247, p = 0.022). However, the rate of improvement did not vary across the two conditions (t-ratio = 0.930, df = 97, p = 0.355). Significant effects were found for fewer baseline psychiatric symptoms (t-ratio = −2.49, df = 97, p = 0.015); previous paid employment (t-ratio = 3.85, df = 97, p < 0.001); having received additional support (t-ratio = 2.39, df = 97, p = 0.019); and supported and sheltered housing (t-ratio = −2.14, df = 97, p = 0.035). Although the per-protocol analysis (n = 119) showed a significant increase in SFS_OE scores during the study period, and a significant effect for symptoms and previous paid employment, it showed no significant effect for condition, and no effect for additional support or supported and sheltered housing.

**Table 3:** Multilevel model including possible confounders for the primary outcome measures.

	SFS_OE			Participation ladder		Hours of participation^19^	
Variable	Coefficient (SE)	OR (95% CI)^20^	P	Coefficient (SE)	P	Coefficient (SE)	P
Intercept	1.169 (0.955)	3.219 (0.481–21.539)	0.244	3.636 (0.306)	<0.001***	7.483 (2.069)	<0.001***
Time	0.520 (0.226)	1.682 (1.077–2.628)	0.022	0.169 (0.063)	0.008**	1.397 (0.492)	0.005**
Condition	0.661 (0.711)	1.936 (0.472–7.936)	0.355	0.006 (0.215)	0.978	0.892 (1.373)	0.518
Time*condition	−0.364 (0.305)	0.695 (0.381–1.267)	0.234	-0.012 (0.088)	0.893	−0.499 (0.668)	0.456
Baseline level of psychiatric symptoms	−0.041 (0.016)	0.969 (0.930–0.992)	0.015*	-0.019 (0.005)	<0.001***	−0.150 (0.042)	<0.001***
Previous work experience (yes -no)	2.388 (0.620)	10.895 (3.184–37.282)	<0.001***	0.743 (0.176)	<0.001***	5.507 (1.554)	<0.001***
Additional vocational inputs (yes – no)	1.173 (0.491)	3.231 (1.220–8.558)	0.019*	0.306 (0.156)	0.053	3.504 (1.265)	0.007**
Supported/sheltered housing (yes – no)	−1.038 (0.485)	0.354 (0.135–0.928)	0.035*	−0.014 (0.157)	0.929	0.185 (0.969)	0.849
Practitioner work experience in years	−0.019 ()0.019	0.981 (0.945–1.019)	0.321	−0.001 (0.006)	0.919	0.072 (0.045)	0.115
Model fit							
R^2^ (R^2^ per-protocol) ICC_subject_ (ICC_subject_ per-protocol) ICC_practitioner_ (ICC_practitioner_ per-protocol)	0.204 (0.20) 0.621 (0.60) 0.000 (0.00)			0.140 (0.20) 0.498 (0.51) 0.001 (0.00)		0.150 (0.18) 0.553 (0.56) 0.001 (0.00)	

*P < 0.05, **P < 0.01, ***P < 0.001.

During the study period, total hours of participation increased significantly (t-ratio = 2.84, df = 241, p = 0.005), with no difference between the conditions **(**t-ratio = 0.649, df = 97, p = 0.518). There were significant effects for fewer baseline psychiatric symptoms (t-ratio = −3.55, df = 97, p < 0.001); previous paid employment (t-ratio = 3.54, df = 97, p < 0.001); and having received additional support (t-ratio = 2.77, df = 97, p = 0.007). The per-protocol analysis showed similar results and also a positive effect for practitioners with more years of work experience.

Over the study period, scores on the six steps of the participation ladder showed a significant improvement in social participation (t-ratio = 2.67, df = 247, p = 0.008). Again, the rate of improvement did not vary between the two conditions **(**t-ratio = 0.028, df = 97, p = 0.978). There were significant effects for fewer baseline psychiatric symptoms (t-ratio = −3.66, df = 97, p < 0.001) and previous paid employment (t-ratio = 4.22, df = 97, p < 0.001). The per-protocol analysis showed similar results.

### Multilevel Analyses of Secondary Outcome Measures

The rate of improvement did not differ between the conditions for any of the secondary outcome measures (see **Supplementary Table 1**). After 12 months, 43.1% of goals had been fully attained (BPR 43.9%; ACC 42.2%), while 54.8% had not, or had been attained only in part (BPR 54.1%; ACC 55.6%). The percentages of goals that had been fully attained differed between goal areas, but without differences between conditions: paid employment 31% [*χ*(2) = 1.59, p = 0.451]; unpaid employment 66% [*χ*(3) = 2.49, p = 0.477]; education 35.5% [*χ*(1) = 0.53, p = 0.465]; and daily activities and social contact 52.9% [*χ*(1) = 1.02, p = 0.312]. Goal attainment was significantly influenced by fewer baseline psychiatric symptoms (t-ratio = −3.59, df = 94, p < 0.001) and by previous paid employment (t-ratio = 2.25, df = 94, p = 0.027). The model with the per-protocol group failed to reach convergence.

QOL improved significantly during the study period (t-ratio = 3.32, df = 237, p = 0.001), with significant effects for fewer baseline psychiatric symptoms (t-ratio = −5.18, df = 97, p < 0.001), previous paid employment (t-ratio = −2.99, df = 97, p = 0.004) and for practitioners with less work experience in MHC (t-ratio = 2.25, df = 94, p = 0.027). The results of the per-protocol analysis were comparable.

Change in personal recovery was significantly influenced by fewer baseline psychiatric symptoms (t-ratio = −6.12, df = 97, p < 0.001). The per-protocol analysis showed similar results.

Change in self-efficacy was significantly influenced by fewer baseline psychiatric symptoms (t-ratio = −4.19, df = 97, p < 0.001). The per-protocol analysis showed similar results.

Psychosocial functioning improved significantly during the study period (t-ratio = 2.35, df = 236, p = 0.020), with significant effects for fewer baseline psychiatric symptoms (t-ratio = −3.75, df = 97, p < 0.001) and previous paid employment (t-ratio = 2.47, df = 97, p = 0.015). While the per-protocol analysis showed similar results, it did not show the significant improvement in psychosocial functioning over the study period.

## Discussion

In our study, BPR did not improve social participation more effectively than ACC. Social participation, QOL, and psychosocial functioning improved in both groups during the study period, with around 43% of the participants per group attaining their social participation goals. Our finding that previous working experience and baseline level of psychiatric symptoms consistently predicted outcome is in line with studies on predictors for vocational functioning in individuals with SMI (37). The overall explained variance was low (<20%), indicating that the multilevel models were also influenced by factors we had not investigated.

As in previous studies on the effectiveness of BPR (14, 15), most participants had goals with regard to paid employment and education, and fewer of them chose either unpaid work or daily activities and social contacts. Most of those who changed their goals changed from paid employment to unpaid employment or other meaningful daily activities. Similarly, in ACC and BPR and alike, the percentages of those who fully attained their goals with regard to paid employment and education were lower than in the other goal areas.

Our study is not the first in which the effect of the experimental condition was no greater than that of the control. Like us, Rogers et al. (18) found that both groups improved, but that BPR was not superior to ACC in the vocational domain. They suggested that this may have been due to various improvements that had been made to ACC in order to prevent dropout. If so, it may be possible to attribute the small difference between the conditions in our own study to the fact that practitioners in ACC called in help from specialist vocational services —including IPS employment specialists— significantly more often than BPR practitioners did, even though the involvement of additional inputs was allowed in both conditions.

It is also possible that, in recent years, the ACC had become more focused on rehabilitation. Although ACC practitioners had not received the same training as BPR practitioners, they may have been influenced by the growing awareness of the importance of helping patients gain employment or other meaningful daily activities (38). To illustrate this possible period effect, we can compare our results with those of a study conducted by members of our research team between 2005 and 2008 on the broad effectiveness of BPR (14), since when MHC facilities and training institutes have paid increasing attention to rehabilitation. In the study in question, goal attainment in BPR after 12 months was approximately twice as high as in ACC. Some 10 years later, as the current study shows, it was comparable. However, in the whole sample in the earlier study, 26.6% of social participation goals had been attained after 12 months, against 43.1% in the current study. Our finding of an overall increase in goal attainment after 12 months may indicate that the greater attention paid to rehabilitation and personal goal attainment is paying off.

As program fidelity was insufficient in one-third of the BPR processes, the absence of a difference in effect may also be explained partly by poor implementation or by program drift. However, the per-protocol analysis, which only included practitioners with sufficient BPR program fidelity, also showed no effect for condition. The explanation may lie in the inclusion of BPR processes whose criterion for BPR fidelity had been set relatively low. As the per-protocol subgroup was very small, it may also lie in a lack of power.

It has also been suggested that BPR is particularly difficult to implement because its operationalization is very complex and may not be easy for MHC professionals from all professional and training backgrounds (21, 39). As poor implementation seems to be a recurring problem in trials of rehabilitation methodologies (40, 41), more effective implementation strategies are needed.

Another possible reason for the lack of an effect of condition in this study is that almost half the participants wanted paid employment, the goal that proved the hardest to attain. The strength of BPR is that it focuses on all rehabilitation-goal areas, which makes it suitable for individuals who are not satisfied with their life in certain domains and wish to explore the options for change, or for those who find it difficult to initiate change. However, due to its broad perspective, BPR is not designed to help people to attain goals such as paid employment, which would then require specific expertise it could not support. More specialized methods may be needed for specific goal areas, such as IPS (42), which is specifically designed to help individuals gain and maintain paid employment and is widely available in the Netherlands (43).

Finally, during the study period, a new social support act was introduced in the Netherlands that brought extensive changes to local government and MHC institutions. These also led to changes to individual recipients, such as cuts in their budgets for meaningful daily activities. New rules also came into effect concerning the permissible types and intensity of support.

We should add that our study was conducted in a period of economic recession. While almost half the participants wanted paid employment, there were few job opportunities. When almost no paid jobs are available, especially for people who are difficult to fit into the labor market, it may not really matter what sort of support is available, as their goals are generally difficult to attain. In such cases, the potential added value of targeted psychiatric rehabilitation approaches is smaller.

An unexpected finding of our study concerned the difference between patients and practitioners with regard to the 6-month ratings of the quality of the therapeutic relationship. While our finding that patients in BPR gave higher ratings than those in ACC may be explained by the person-centered approach of BPR (44), it is unclear why practitioners in ACC gave higher ratings to the relationship with their patients than those in BPR.

The fact that aspects of the therapeutic relationship are important predictors of the effectiveness of psychiatric rehabilitation methods, in general, was shown in a study conducted by members of the current study group (45). The results of that study showed that agreement on goals between practitioner and patient significantly predicted goal attainment at 24 months, for the total study group (BPR and ACC together). Furthermore, goal attainment significantly predicted QOL at 24 months. In that study, BPR was found to be more effective than ACC independent of the effect of agreement on goals. This suggests that the effectiveness of targeted psychiatric rehabilitation approaches such as BPR is also influenced by methodology-specific aspects. However, no studies have been conducted on the working mechanisms of BPR, and this is highly recommended for future research. Furthermore, a recent meta-analysis showed that psychiatric rehabilitation approaches such as BPR could be improved by combining them with cognitive training (46). This is particularly the case in the area of social participation. This notion should be further explored in future research. With regard to goals, several tools have been developed that may aid in clarifying the patients’ goals and support collaborative goal setting. These are the 2-COM (2-way communication), GAS (goal attainment scaling), and CASIG (Clients Assessment of Strengths, Interests, and Goals) (47–49). Perhaps, incorporating these kinds of tools into psychiatric rehabilitation practice could further improve psychiatric rehabilitation effectiveness.

Our study has four main strengths. The first is the heterogeneous group of individuals with SMIs, which made it easier to generalize our results. Second, to the best of our knowledge, this was the first study to include patients with severe long-term eating disorders, whose impairments with regard to social participation are similar to those in people with other SMIs (50). The other strengths are the low attrition rate and the active control group, which ensured that both conditions received equal amounts of attention.

A limitation of our study was the short follow-up period. In the study by Swildens et al. (14), which used a 24-month follow-up period, the rate of goal attainment almost doubled between month 12 and month 24. More time may have been needed to attain social participation goals, particularly during an economic recession, but unfortunately a lack of financial resources did not allow longer follow-up. A second limitation is that fidelity ratings were obtained for only a selection of the BPR practitioners. A third limitation is that although service users were involved in the development of BPR and the design of the study, they were not consulted on the models that were analyzed. As a consequence, their views on the strengths and weaknesses of the studied models are lacking, while these views may have provided relevant clues as to which factors could influence social participation. Although we found no effect for condition in this study, it should be noted that a significant proportion of participants improved their social participation during the study period. This suggests that working on social participation does indeed have the intended effect, irrespective of the specific methodology used. More specifically, in a naturalistic study that monitored employment in FACT teams lacking specialized vocational services, Kortrijk et al. (51) found at one-year follow-up that only 3.9% of individuals with SMI had found paid employment. That is considerably less than the 31.3% in our study and highlights the successes that can be achieved by working on social participation. However, as shown by our finding that less than half of the social participation goals in our study were fulfilled, there is still ample room for improvement.

## Data Availability Statement

The datasets presented in this article are not readily available because Altrecht Mental Health Care needs to consent to data access. Requests to access the datasets should be directed to WS, w.swildens@altrecht.nl.

## Ethics Statement

The studies involving human participants were reviewed and approved by the Research Ethics Committee of the University Medical Center Groningen. The patients/participants provided their written informed consent to participate in this study.

## Author Contributions

SS, WS, JW, and JB formulated the research questions, designed the study, recruited participants, designed and carried out the analysis, and wrote the article. BS recruited participants, coordinated data, and reviewed the manuscript. MM advised on the statistical analyses and reviewed the manuscript. TF, AA, and UD reviewed the manuscript.

## Funding

This research was supported by a grant from the Netherlands Organization for Health Research and Development (ZonMw) (project number 837002006).

## Conflict of Interest

The authors declare that the research was conducted in the absence of any commercial or financial relationships that could be construed as a potential conflict of interest.

## Footnotes

1 Brief Psychiatric Rating Scale (BPRS); scores range from 24–168; higher scores indicate more psychiatric symptoms

2 Global Assessment of Functioning Scale – Symptoms (GAF-S); scores range from 0–100; lower scores indicate more symptoms

3 Global Assessment of Functioning Scale – Disabilities (GAF-D); scores range from 0–100; lower scores indicate more severe disabilities

4 OECD, European Union, UNESCO Institute for Statistics. ISCED 2011 Operational Manual: Guidelines for classifying National Education Programmes and Related Qualifications. OECD publishing, 2015 (36).

5 ISCED level 0, 1, and 2

6 ISCED level 2, 4, and 5

7 ISCED level 5, 7, and 8

8 dichotomized score on the Occupation and Employment subscale of the Birchwood Social Functioning Scale: unemployed (0–6) vs. employed (>= 7); scores range from 0–10; higher scores reflect more social participation

9 Dutch National Societal Participation Ladder; scores range from 1–6; higher scores indicate more social participation

10 Based on those with paid employment

11 Based on those with unpaid employment

12 Manchester Quality of Life Schedule (MANSA); scores range from 12–84; higher scores indicate better QOL

13 Recovery Assessment Scale (RAS); scores range from 41–205; higher scores indicate a higher degree of recovery

14 General Self-efficacy Scale (GSES); scores range from 10–40; higher scores indicate greater self-efficacy

15 Birchwood Social Functioning Scale (SFS) total score; scores range from 0–223; higher scores indicate fewer impairments

16 Not including additional support

17 Helping Alliance Scale – patient version (HAS-P); scores range from 0–52; higher scores indicate better helping alliance

18 Helping Alliance Scale – therapist version (HAS-T); scores range from 0–50; higher scores indicate better helping alliance

19 Because residuals were slightly skewed, we report robust standard errors here

20 Odds ratios and confidence intervals are provided for dichotomous outcomes

## Supplementary Material

Supplementary Table 1 Multilevel model including possible confounders for the secondary outcome measures.
