# Supplementary material for: Effectiveness of the Boston University Approach to Psychiatric Rehabilitation in Improving Social Participation in People With Severe Mental Illnesses: A Randomized Controlled Trial
Source: Front Psychiatry. 2020 Sep 23;11:571640. doi: 10.3389/fpsyt.2020.571640 (PMC7538503; doi:10.3389/fpsyt.2020.571640)
Supplement: Supplementary Table 1 — Multilevel model including possible confounders for the secondary outcome measures. [file Table_1.docx]

Supplementary table 1. Multilevel model including possible confounders for the secondary outcome measures

|  | Goal |  |  | QOL |  | Personal recovery |  | Self-efficacy |  | Psychosocial functioning |  |
| --- | --- | --- | --- | --- | --- | --- | --- | --- | --- | --- | --- |
| Variable | Coefficient (SE) | OR (95% CI) | P | Coefficient (SE) | P | Coefficient (SE) | P | Coefficient (SE) | P | Coefficient (SE) | P |
| Intercept | 0.980 (0.525) | 2.665 (0.937-7.579) | 0.066 | 68.867 (3.445) | <0.001*** | 101.686 (3.539) | <0.001*** | 33.872 (1.636) | <0.001*** | 146.925 (6.474) | <0.001*** |
| Time | 0.090 (0.163) | 0.988 (0.970-1.005) | 0.579 | 1.652 (0.498) | 0.001 | 0.775 (0.511) | 0.131 | 0.043 (0.230) | 0.851 | 1.844 (0.786) | 0.020 |
| Condition | 0.071 (0.482) | 1.074 (0.412-2.799) | 0.882 | -0.531 (2.042) | 0.796 | -1.750 (2.096) | 0.406 | -1.556 (0.961) | 0.109 | -0.965 (3.628) | 0.791 |
| Time*condition | -0.031 (0.225) | 0.969 (0.622-1.511) | 0.891 | -0.486 (0.689) | 0.481 | -0.043 (0.705) | 0.952 | 0.545 (0.318) | 0.088 | -0.985 (1.087) | 0.366 |
| Baseline level of psychiatric symptoms | -0.029 (0.008) | 0.972 (0.957-0.987) | <0.001*** | -0.316 (0.061) | <0.001 | -0.382 (0.062) | <0.001 | -0.122 (0.029) | <0.001 | -0.434 (0.116) | <0.001 |
| Previous work experience (yes – no) | 0.570 (0.254) | 1.769 (1.069-2.927) | 0.027 | 5.210 (2.089) | 0.014 | 2.910 (2.140) | 0.177 | 1.560 (0.994) | 0.120 | 9.842 (3.982) | 0.015 |
| Additional vocational inputs  (yes – no) | 0.376 (0.223) | 1.456 (0.935-2.267) | 0.095 | 0.0126 (1.853) | 0.995 | -0.415 (1.897) | 0.827 | -0.142 (0.882) | 0.873 | 5.772 (3.532) | 0.105 |
| Living in supported/sheltered housing (yes – no) | -0.212 (0.227) | 0.809 (0.516-1.270) | 0.353 | -1.018 (1.862) | 0.586 | 1.584 (1.910) | 0.409 | 0.430 (0.885) | 0.628 | -6.918 (3.549) | 0.054 |
| Practitioner work experience in years | -0.012 (0.009) | 0.988 (0.970-1.005) | 0.172 | -0.214 (0.072) | 0.004 | -0.0115 (0.074) | 0.876 | 0.015 (0.034) | 0.669 | -0.140 (0.136) | 0.306 |
|  |  |  |  |  |  |  |  |  |  |  |  |
| Model fit  R^2^ (R^2^ per-protocol)  ICC_subject_ (ICC_subject_ per-protocol)  ICC_practitioner_ (ICC_practitioner_ per-protocol) | 0.075 (†)  † (†)  † (†) |  |  | 0.186 (0.18)  0.704 (0.77)  0.033 (0.00) |  | 0.167 (0.22)  0.740 (0.76)  0.002 (0.01) |  | 0.099 (0.11)  0.731 (0.71)  0.001 (0.00) |  | 0.146 (0.20)  0.800 (0.81)  0  .010 (0.00) |  |

* P < 0.05, **P < 0.01, ***P < 0.001

† Because the null model could not be estimated with adaptive Gaussian iterations, ICC could not be calculated
